# AutoPlate: Rapid Dose-Response Curve Analysis for Biological Assays

**DOI:** 10.3389/fimmu.2021.681636

**Published:** 2022-02-10

**Authors:** Phil Palmer, Joanne Marie M. Del Rosario, Kelly A. S. da Costa, George W. Carnell, Chloe Q. Huang, Jonathan L. Heeney, Nigel J. Temperton, David A. Wells

**Affiliations:** ^1^Laboratory of Viral Zoonotics, Department of Veterinary Medicine, University of Cambridge, Cambridge, United Kingdom; ^2^Viral Pseudotype Unit, Medway School of Pharmacy, University of Kent, Chatham, United Kingdom; ^3^Department of Physical Sciences and Mathematics, College of Arts and Sciences, University of the Philippines Manila, Manila, Philippines; ^4^DIOSynVax, University of Cambridge, Cambridge, United Kingdom

**Keywords:** pseudotype neutralization, ELLA, data analysis, biological assay, dose-response, pseudovirus, SARS-C0V-2, influenza

## Abstract

The emergence of COVID-19 has emphasised that biological assay data must be analysed quickly to develop safe, effective and timely vaccines/therapeutics. For viruses such as SARS-CoV-2, the primary way of measuring immune correlates of protection is through assays such as the pseudotype microneutralisation (pMN) assay, thanks to its safety and versatility. However, despite the presence of existing tools for data analysis such as PRISM and R the analysis of these assays remains cumbersome and time-consuming. We introduce an open-source R Shiny web application and R library (AutoPlate) to accelerate data analysis of dose-response curve immunoassays. Using example data from influenza studies, we show that AutoPlate improves on available analysis software in terms of ease of use, flexibility and speed. AutoPlate (https://philpalmer.shinyapps.io/AutoPlate/) is a tool for the use of laboratories and wider scientific community to accelerate the analysis of biological assays in the development of viral vaccines and therapeutics.

## Introduction

The pseudotype based microneutralisation (pMN) assay measures functional antibody responses against viruses (1). The pMN assay uses pseudotypes or pseudoviruses, viral vectors which usually display the envelope protein of the virus of interest on its surface with a marker or reporter gene, commonly luciferase or green fluorescent protein (2, 3). The viral entry protein or envelope enables the pseudotypes to enter cells which then express the encoded marker allowing viral entry to be quantified. Antibodies that inhibit virus entry will reduce the expression of the pseudotype marker and so the potency of neutralisation can also be quantified (1, 4).

**Figure 1:**
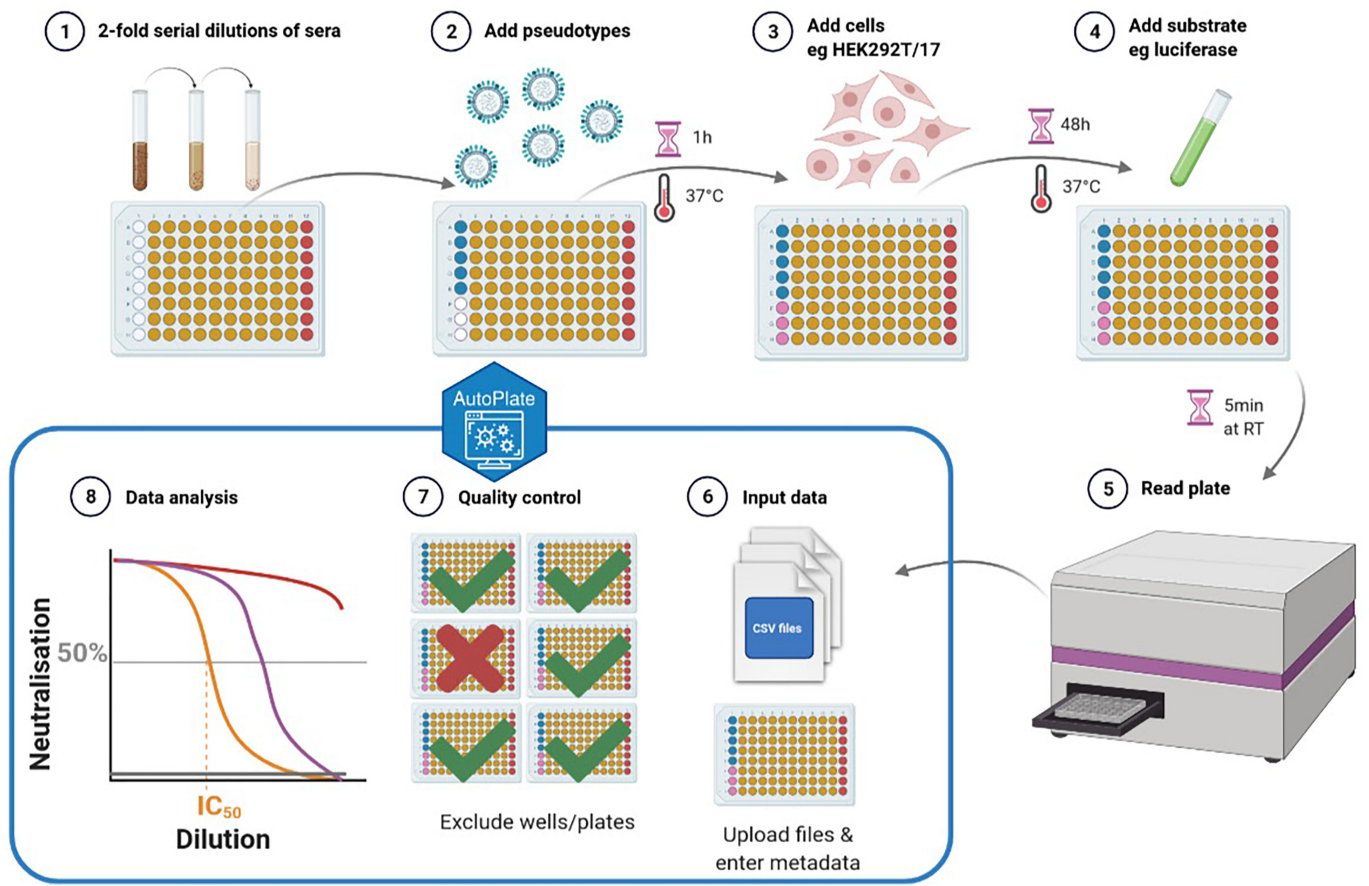
Schematic representation of the pseudotype neutralisation (pMN) assay. Steps 1-5 show the lab protocol for the pMN assay and steps 6-8 show the computational analysis using AutoPlate. The plate layout for the well types can be seen where the sera (type “x”) are shown in yellow, antibody positive control (type “m”) shown in red, virus (type “v”) shown in blue and cells (type “c”) shown in pink (created with BioRender) (6).

Neutralisation potency is usually reported as the IC_50_ of a dose-response curve. By measuring neutralisation along a dilution series, a dose-response curve can be estimated for a given antibody/sera (1). When this dilution is displayed on a log scale the curve follows a classic S shape which is well represented by the 4-parameter log-logistic regression curve (5). As shown by **Figure 1**, the IC_50_ is defined as the dilution which gives 50% neutralisation of the curve (1, 5).

Influenza pseudotypes are predominantly used for the pMN assays but can also be used for the enzyme-linked lectin assay (ELLA) that measure inhibition of neuraminidase (NA) activity (7). Neuraminidase is the second most abundant influenza surface glycoprotein after haemagglutinin (HA). ELLA can also be performed using 96-well plates in almost the same format as pMN, with the response shown *via* dose-response curves and reported as IC_50_ values (8).

There are three major advantages of pMN and pELLA (pseudotype based ELLA) compared to other biological assays (bioassays) for measuring immune response against viruses. These assays are very safe (1, 2, 9), versatile (2, 3), as they can be used for a range of viruses, and have growing adoption for emerging viruses (3, 10, 11).

The assays are safe because the pseudotypes used are replication-incompetent meaning that they cannot replicate as they do not contain all the genes from the original viral vector (most commonly a lentivirus or retrovirus) needed to replicate (1, 2). As a result, these assays can be performed at a lower biosafety level (BSL) (3, 9, 11). For example, SARS-CoV-2 pMN can be performed in BSL 2 laboratories but live SARS-CoV-2 requires BSL 3 facilities, further increasing the speed at which vaccines and other therapeutics can be developed (4, 9, 12).

The pMN assay can be applied to virtually any enveloped virus as it measures cell entry rather than a specific feature of the virus (2). pMN has been applied to many viruses including influenza (1, 12–15), HIV (16, 17), Ebola (18, 19), MERS (9, 20), Dengue (21), Lassa (22), Rabies (23), Chikungunya (24) and Nipah virus (25). It has become one of the principal assays for characterising functional immune response during the ongoing SARS-CoV-2 pandemic (4, 12, 26), which further indicates its rapid uptake and applicability to new and emerging viruses (3, 10).

Once the experiment has been run the two main steps to analyze it are reformatting the data and statistical analysis (1). Although there are proprietary and open-source tools for the analysis there are drawbacks to currently available software solutions and the time-consuming reformatting is not handled by either.

The main input for the computational analysis of the immunoassays is raw luminescence (or fluorescence) data, often contained within tabular files (normally CSV or Excel) that specify relative luminescence units (RLU) values for each well (1). However, the crucial experimental metadata is usually not included and so must be carefully entered for each well. Along with reformatting the data to be entered into the chosen stats package, this is the most time-consuming step of the computational analysis and where an intuitive and efficient interface could most benefit labs running these assays.

## Results

### AutoPlate

We present AutoPlate as a simple interface to quickly add experimental metadata to immunoassay results, reformat data and perform statistical analysis. AutoPlate produces publication-ready figures but allows users to export data for further analysis with external statistical software such as R. AutoPlate can be accessed through an online Shiny app or installed as an R package. The AutoPlate source code is open source and available at https://github.com/PhilPalmer/AutoPlate.

### How Does AutoPlate Compare to Other Existing Software?

Existing proprietary software such as PRISM allows for the analysis of bioassays *via* a graphical user interface (GUI) (1). This helps make it easier to enter data, however, it is rigid compared to tools such as the open-source R and Python programming languages and there is little/no integration with these languages. The R and Python programming languages have software packages “drc” and “neutcurve” respectively (5, 27). These packages are incredibly flexible for dose-response curve analysis but require a technical understanding of their respective programming languages (5). Crucially, preparing data for analysis is slow in all programs especially when analysing many 96-well plates, as shown in **Table 1**.

**Table 1:** Qualitative comparison between AutoPlate and currently available software for analysing data from bioassays.

Tool	Graphical user interface (GUI) available? (Ease of Use)	Command line software package available? (Flexibility)	Handles reformatting of raw plate data? (Data Entry Speed)
AutoPlate	Yes	Yes	Yes
PRISM	Yes	No	No
R (drc)	No	Yes	No
Python (neutcurve)	No	Yes	No

### Overview of the Application

AutoPlate provides an intuitive graphical user interface for quickly adding experimental metadata and formatting the data for analysis. This formatted data can be exported for analysis in other software or analysed within AutoPlate to produce a report including quality control checks and publication-quality figures. AutoPlate is implemented in the R Shiny framework (https://shiny.rstudio.com/) because it combines the flexibility of R (with packages such as “drc”) with a user-friendly graphical user interface (similar to PRISM). Analysing data using AutoPlate follows three steps.

#### Step 1) Input

**Figure 2:**
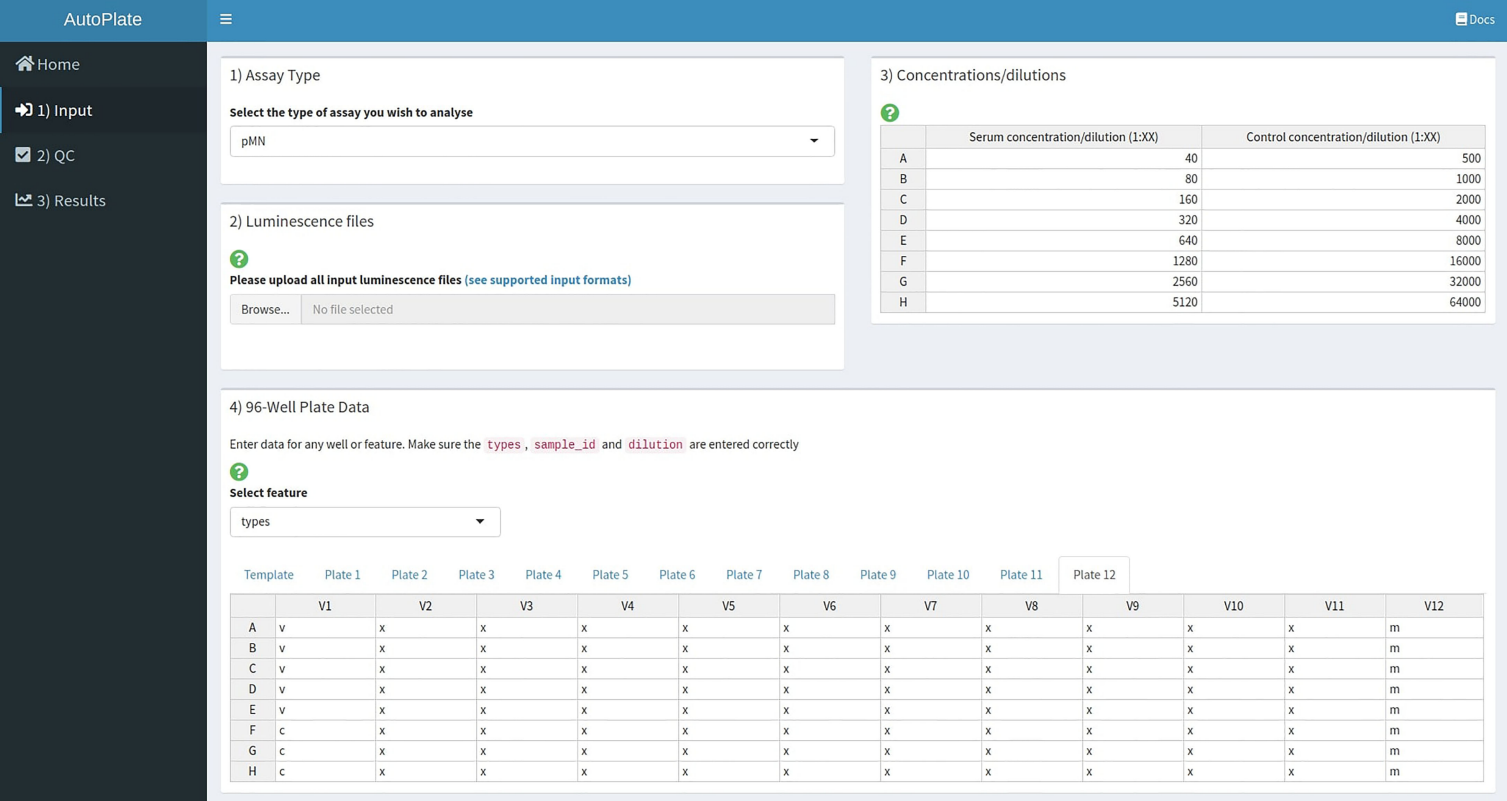
AutoPlate Step 1) Input Screenshot. Upload raw data from the plate reader and specify metadata required for dose-response curve analysis.

**Figure 3:**
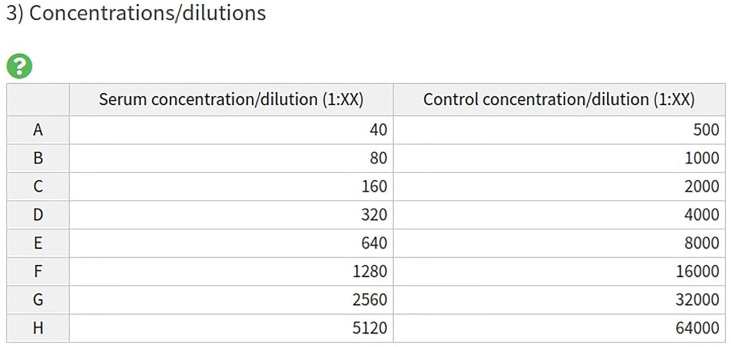
AutoPlate Step 1) Input Concentrations/Dilutions Table Screenshot. Specify the serum and control concentrations/dilutions.

To add experimental metadata before data analysis AutoPlate accepts five inputs (see **Figure 2**):

1) The assay type is defined, currently supported assays are the pMN and ELLA assays.
2) Luminescence files that contain the raw luminescence values for each well can be uploaded directly from the plate reader. CSV format is used for the pMN assay and Excel format for the ELLA assay. Users can also upload a previously exported CSV file generated from a later step in AutoPlate if they want to re-load a dataset that has already been analysed.
3) Both the serum and control concentration/dilutions can be specified by an interactive table, containing default values (**Figure 3**). Each row in the table corresponds to rows or columns on the 96-well plate for the pMN and ELLA assays respectively.
4) For each uploaded 96-well plate, an 8x12 interactive table will be generated. This table can be used to view and change the values for each well for any feature such as the sample types, treatment group or sample ID, etc., by selecting that feature from the drop-down menu. Initially, wells are populated with default values commonly used in our lab for the types, sample IDs and dilutions. For example, for the pMN assay, two columns are used per sample with the sample ID increasing across the plate(s), with the first and last columns being reserved for the controls. Modifications can be made in bulk by modifying the “template” to allow any plate layout to be quickly propagated across all plates.

**Figure 4:**
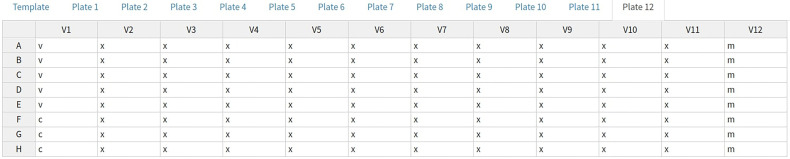
AutoPlate Step 1) Plate Data Table Screenshot. Enter data for any plate, well or feature such as the well type.

The default pMN types layout can be seen in **Figure 4**. Most wells are type “x” (experimental); these are a known dilution of sample neutralizing the virus of interest. Type “m” indicates the positive control (“m” was originally for monoclonal antibodies), these wells are treated the same as type “x” but can be easily filtered or colored differently and may be diluted differently. The final types are “v” and “c” which are used to convert virus marker measurements into neutralisation and normalize the data. Type “c” contains cells only and represents 100% neutralisation because there is no virus to enter cells and express the marker. Type “v” contains cells and virus-only but (no treatment) and represents 0% neutralisation because no virus neutralisation occurs.

5) The final input is any other features such as the bleed, treatment, virus and experiment ID. The bleed is normally an integer corresponding to the week the sample (e.g., mouse) was bled or “terminal” for the last bleed. The treatment will be used to group the data for the dose-response curves, for example, what the subjects were inoculated with. The virus refers to the pseudotyped virus (pseudovirus) that was used in the assay. The experiment ID is a unique identifier specifying which study generated the data. You can have multiple experiment IDs if you are analysing multiple datasets from different experiments. All these features can be set using the 96-well plate data tables or in the “other features” section. This implementation of the other features section was designed to be as flexible as possible, allowing the user to set these features by selecting any existing feature and providing a mapping for a new value. For example, it is often best to set the treatment based upon the sample ID if you are specifying which vaccination each subject received. These four features were chosen as default because they are common to our group. However, these features could be omitted, and other ones added in the future.

#### Step 2) Quality Control

**Figure 5:**
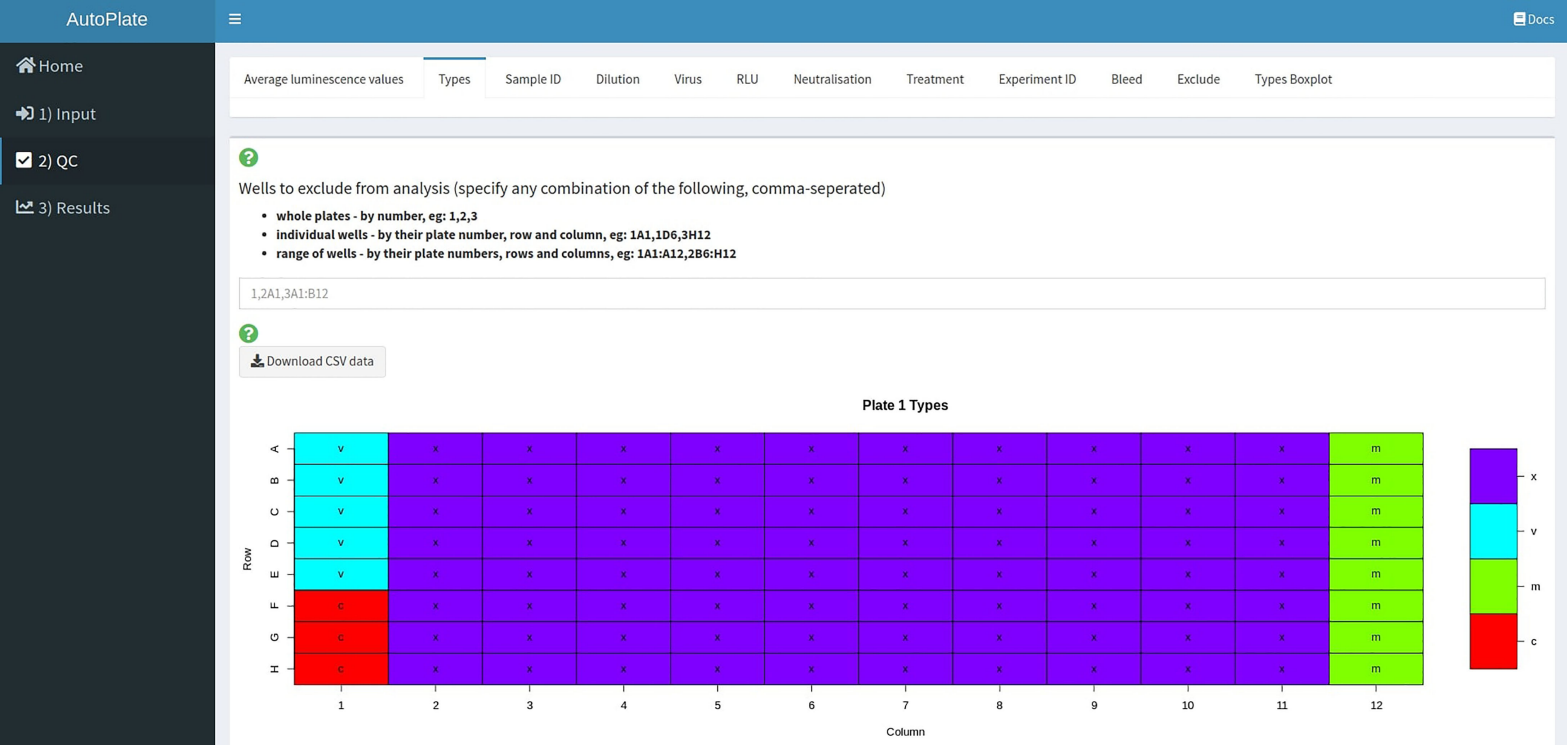
AutoPlate Step 2) Quality Control Screenshot. Visualise the data entered in Step 1 and check that the controls have worked for each plate/well.

The quality control step (**Figure 5**) allows users to quickly determine that the data is entered correctly and that the controls have worked as expected. To visualize the data entered in Step 1 a table, various heatmaps and a boxplot are generated. If the controls for a particular plate or well have failed, then these wells can be excluded from the analysis.

**Figure 6:**
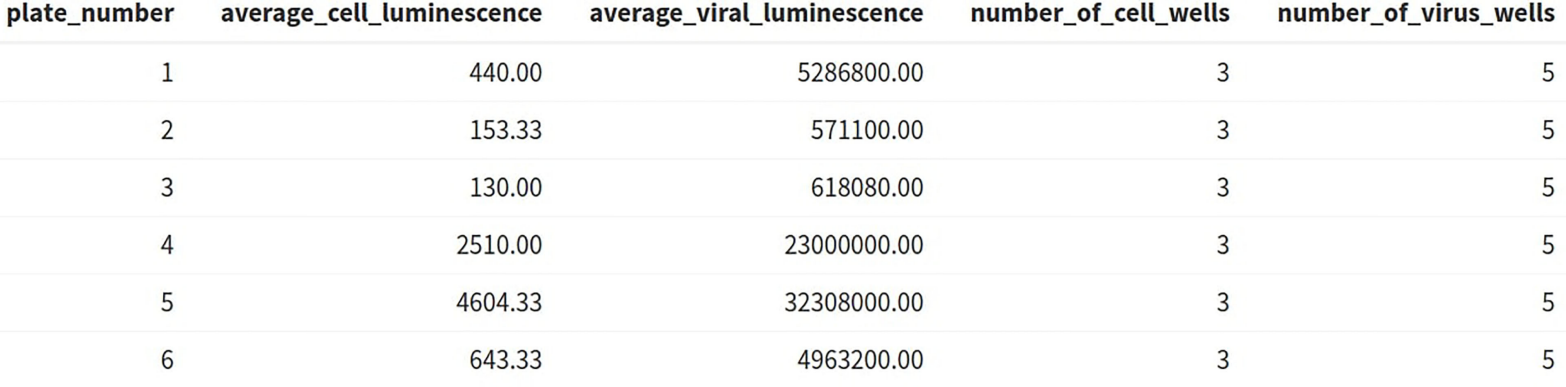
AutoPlate Step 2) Quality Control Average Luminescence Table Screenshot. Check the number of control wells and difference in luminescence values between the virus-only and cell-only control wells.

A table is generated (**Figure 6**) displaying the average viral and cell luminescence to check that there is a substantial difference in the average luminescence between virus-only and cell-only wells and that an adequate number of control wells have been included in the analysis.

**Figure 7:**
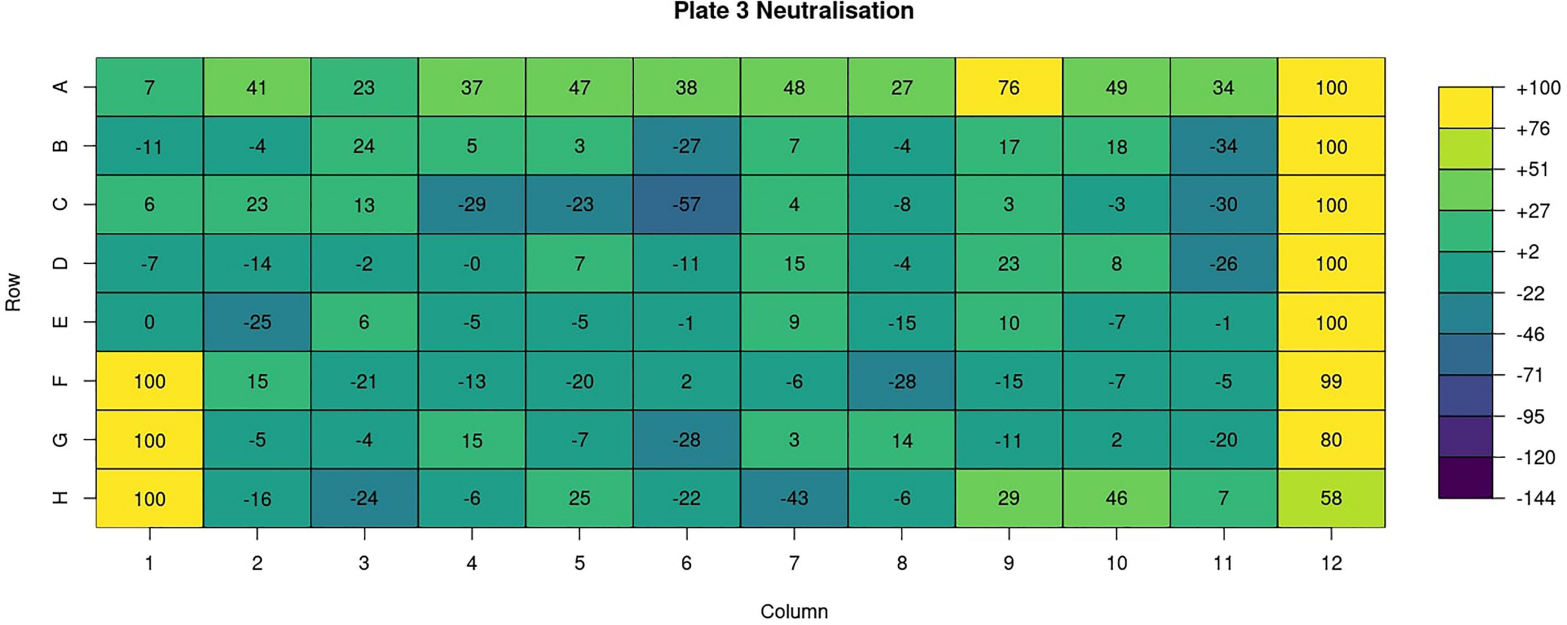
AutoPlate Step 2) Quality Control Neutralisation Heatmap Screenshot. Visualise features such as the neutralisation in a 96-well plate format using a heatmap.

Heatmaps are generated for various key features (well types, sample ID, dilution, virus, RLU, neutralisation (**Figure 7**), treatment, bleed and experiment ID). For all these features a heatmap is used to show the value for each of these features for all plates in a 96-well plate format i.e., an 8 x 12 grid.

A user can choose to exclude any wells from the analysis. To do this they can specify a string consisting of comma-separated values of either whole plates, individual wells or a range of wells. Wells can also be excluded from the plate data table in the input step (**Figure 4**). Wells could be excluded for any number of reasons but are most commonly excluded when the controls have failed or when wells were left empty on a particular plate. A heatmap is also generated to show which wells will be excluded from the analysis, allowing users to verify that the correct wells have been excluded from the analysis.

**Figure 8:**
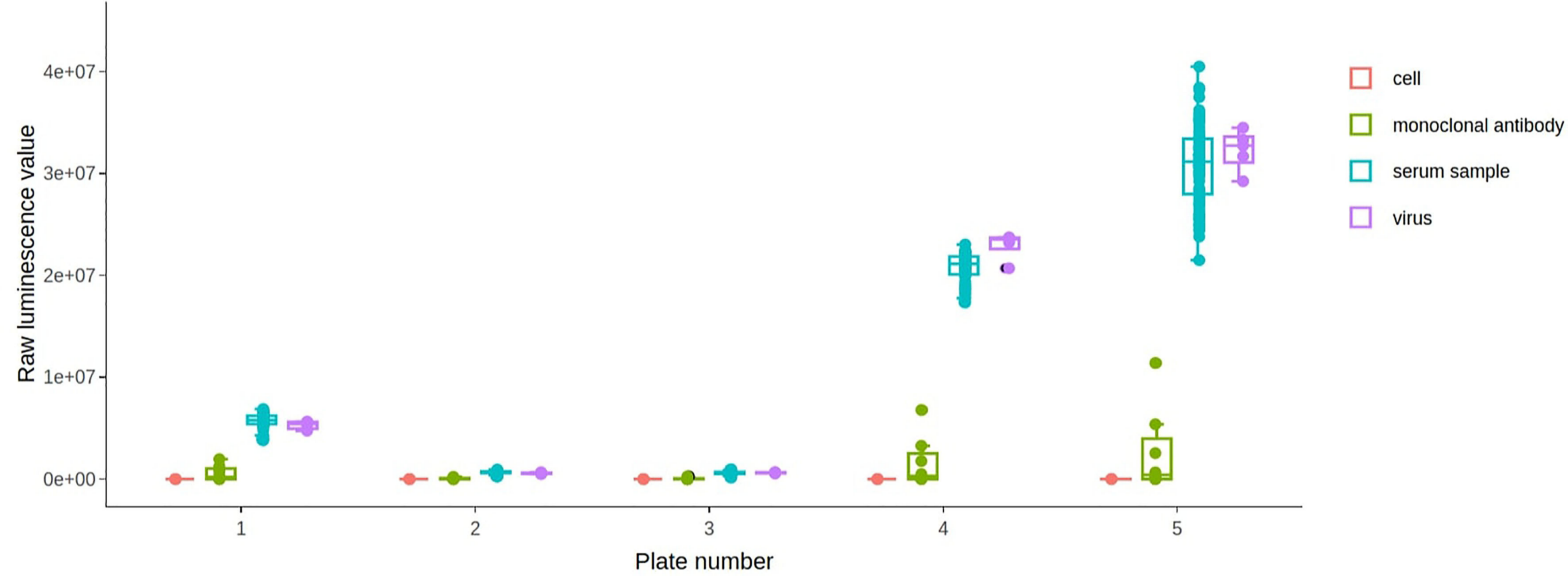
AutoPlate Step 2) Quality Control Types Boxplot Screenshot. The graph shows the average raw luminescence value for each well type on each plate. The legend functions as a toggle to show which well types are shown.

A boxplot of all the different well types for each plate is generated (**Figure 8**). This plot can be used to check differences in control values between plates. It is important to check that the virus wells have approximately the highest luminescence and cell wells have the lowest because it is these wells that are used for the normalization when calculating the neutralisation. In our experience, if the virus-only wells do not have the highest values on a plate, the dose-response curves estimated from that plate are shifted downwards, and this may underestimate the neutralizing capacity of the sample sera.

Users can export the formatted dataset containing all assay data as a CSV from AutoPlate. This allows the data to be shared or analysed with different statistical software. This dataset can also be uploaded on the first step of AutoPlate so that shared data can be analysed in AutoPlate or old data can be reanalyzed.

#### Step 3) Results

**Figure 9:**
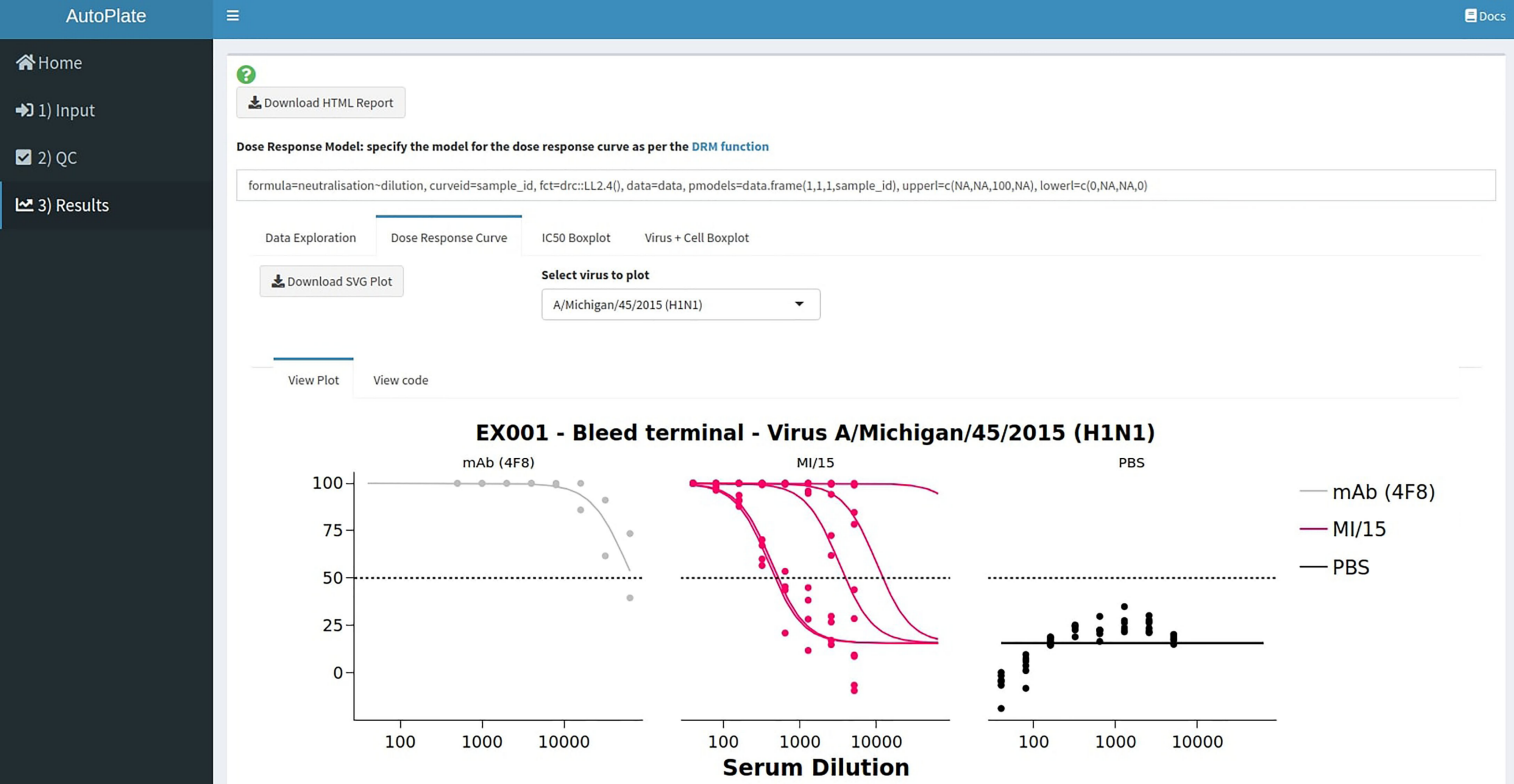
AutoPlate Step 3) Results Screenshot. Fit dose-response curves to analyse the data entered in previous steps, calculate the IC_50_ values and generate downloadable plots to visualise the results.

The following four interactive plots are generated in the results section (**Figure 9**), data exploration, dose-response curve, IC_50_ boxplot and virus-cell boxplot. All these plots can be downloaded as a publication-quality figure in either SVG or PNG format and the raw code required to reproduce the plot in R can be viewed.

**Figure 10:**
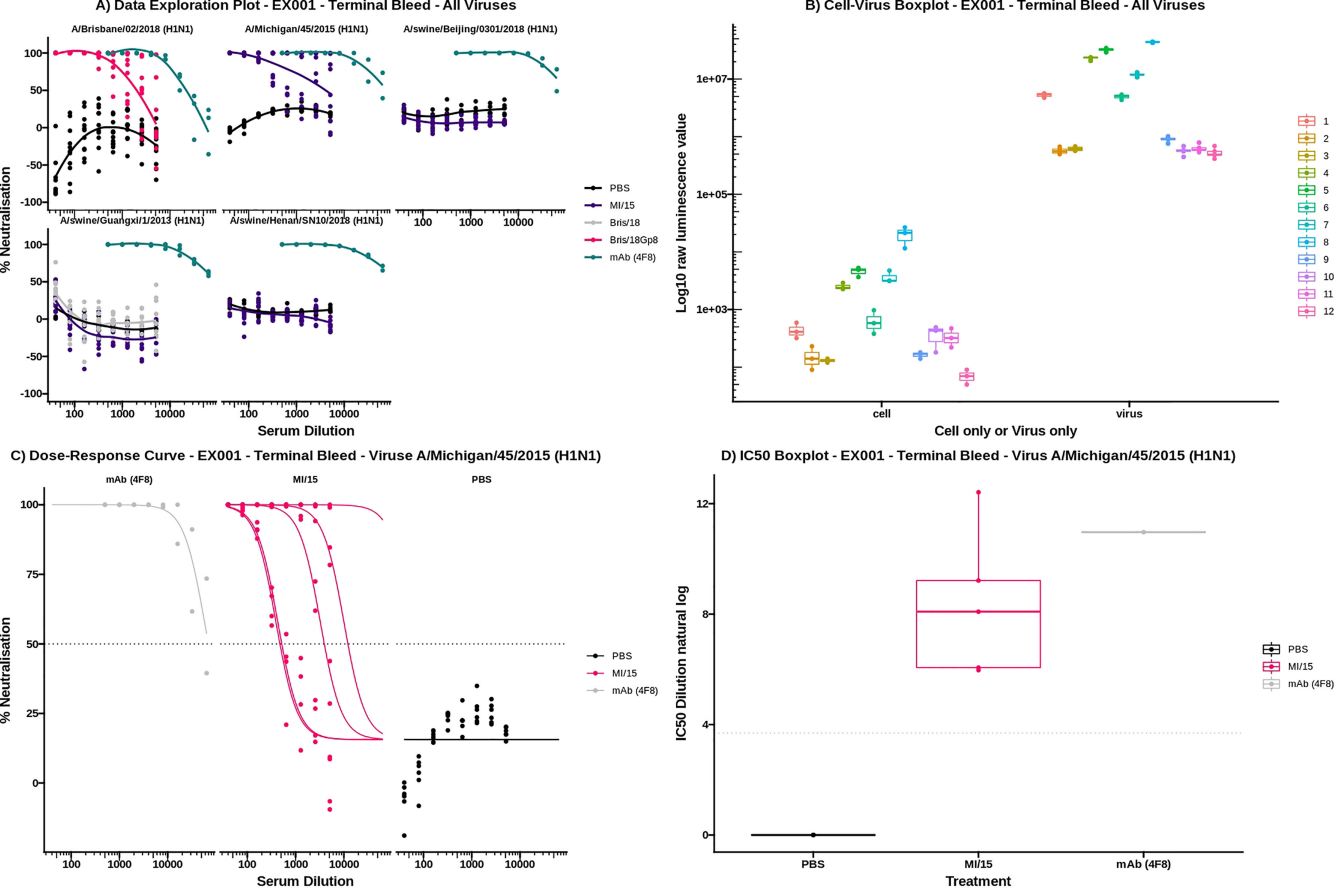
AutoPlate Step 3) Results Plots Screenshot **(A)** Data Exploration Plot. For each virus the serum dilution is plotted on the x-axis against the neutralisation on the y-axis. Each line represents a different treatment group. **(B)** Virus Cell Boxplot. The graph shows cell and virus only well types on the x-axis for each plate plotted against the log raw luminescence value. **(C)** Dose-response Curves Plot. The plot for virus A/Michigan/45/2015 (H1N1) is shown with the serum dilution plotted on the x-axis against the neutralisation on the y-axis. Each line represents a single sample and is coloured by the treatment group. **(D)** IC_50_ Boxplot. The plot for virus A/Michigan/45/2015 (H1N1) is shown with the log IC_50_ dilution plotted on the x-axis against the treatment group on the y-axis. Each data point represents a single sample.

The data exploration plot (**Figure 10A**) fits a loess smooth to each treatment group to illustrate the dose-response relationship of treatment groups outside of the dose-response curve model. The virus-cell boxplot can be used to check that the controls have worked as expected (**Figure 10B**). There should be a clear separation between the virus and cell groups and little variation between plates. The dose-response tab fits a 4-parameter log-logistic regression dose-response model to each of the treatments (**Figure 10C**) (5). To simplify visual comparison of treatments a boxplot of just the IC_50_ values of each curve is displayed (**Figure 10D**).

The dose-response model, which is used for the dose-response curve and IC_50_ box plot, can also be defined, allowing a great deal of flexibility. To produce the plots, the “drc” R package is used and the user can define any parameters to the “drm” function they wish such as the equation, model type, which parameters are shared between treatment groups, and any upper/lower limits for the parameters used by the dose-response model (5).

All the information displayed within AutoPlate, such as the quality control and results plots can be downloaded as a shareable HTML report file. As well as including all the plots generated in AutoPlate, it also contains the specific code and R package versions needed to reproduce them.

### Applications of AutoPlate

To show the speed, flexibility and ease of use of AutoPlate we have provided an example dataset. This dataset was used for all figures, can be loaded within AutoPlate and the raw data can be found within the GitHub link. The dataset consists of five different Influenza H1N1 viral pseudotypes tested against mice inoculated with PBS, A/Michigan/45/2015 (MI/15) and A/Brisbane/02/2018 (Bris/18). The dataset shows a range of neutralisation responses, as shown in **Figure 10**. For all viruses, the negative control (PBS) shows very low neutralisation and the positive control (HoxB8 Antibody, 4F8) shows very high neutralisation. The two inoculates show very low neutralisation except when they are homologous i.e. the strain against which the mice were inoculated is the same as the viral pseudotype, in which case the mice showed moderate neutralisation.

## Discussion

AutoPlate is a tool accessible through a web application or R package, to help further automate the analysis of bioassays. The main objective of AutoPlate was to enable users to go from raw data to publication-quality plots quickly by handling the time-consuming reformatting of data which is not handled by the other tools. This allows considerable time saving and therefore shortens the feedback loop for data analysis, which is especially useful when conducting large high throughput experiments.

We also aimed to make AutoPlate user-friendly. There is no need for the user to have previous programming experience or any sophisticated software installed on their computer, only a web browser, unlike software packages drc and neutcurve. This helps make the data analysis more accessible to the scientist performing the assay, who may have crucial input or valuable domain-specific knowledge.

Golem is a useful tool for developing Shiny applications with software best practices as a standalone R package so that users can run their own version (28). AutoPlate development was supported with Golem including automated testing, extensive documentation and ensuring the package consists mainly of modular R functions. This makes AutoPlate easier to maintain, install and means that users can use AutoPlate as a package rather than a Shiny app.

Finally, AutoPlate is very flexible compared tools such as PRISM, due to its integration with R. It is possible to install AutoPlate as an R library, view the raw R code for all the plots and get a list of all the R packages used including versions from the HTML report. This makes reproducing the analysis within R trivial, allowing the user to tweak or extend the analysis performed with all the options available in R. Any analysis within the AutoPlate web application is reproducible because it is possible to export the full dataset and re-uploading and analysing the same dataset will generate the same results. As AutoPlate is open-source, it is also highly extendable. For example, AutoPlate could support new analyses, new assay types such as ELISA (enzyme-linked immunosorbent assay), HIA (hemagglutination inhibition assay) or even any custom assay and other plate types such as 384-well plates.

In summary, AutoPlate is a fast, easy to use and flexible web application to help accelerate the analysis of biological assay data.

## Methods

AutoPlate was developed using R v3.6.3 and R Shiny v1.5.0 (29, 30). The neutralisation is calculated from the RLU values and normalized per plate. The minimum level of cell infection is calculated as the median luminescence from the virus-only wells, for a given plate. The maximum level of cell infection is calculated as the median luminescence from the cell-only wells, for a given plate. The neutralisation for each well is then expressed as a percentage between the minimum and maximum levels of cell infection as calculated for that plate. This is shown in the equation below for calculating the neutralisation, where x is the RLU for a particular well, v is the median RLU for the virus-only wells for the plate and c is the median RLU for the cell-only wells for the same plate.


$$neutralisation=100\times \frac{x-v}{c-v}$$

Dose-response curves and IC_50_ values were estimated using the R package drc v3.0-1 (5). By default, for the dose-response model, the dose is the dilution, and the neutralisation is the response, the sample IDs are used to group the data. The model used is the four-parameter log-logistic function (or LL2.4) which can be denoted by the expression below, where b is the slope around the IC_50_, c is the curve minimum, d is the curve maximum and e is the log IC_50_ value (5). This model was chosen as the default option because we found that models more readily converge when estimating log(IC_50_) than IC_50_ itself. By default, a single curve minimum, curve maximum, and gradient are estimated for the whole population to improve comparisons between samples. The upper limit of the curve maximum is set to 100 and the lower limit for the gradient around the IC_50_ and the IC_50_ value are set to zero to prevent a negative gradient or IC_50_ value.


$$f(x)=c+\frac{d-c}{1+\exp(b(\log(x)-e))}$$

## Code Availability

The AutoPlate web application can be accessed freely at https://philpalmer.shinyapps.io/AutoPlate/. Users are encouraged to modify, contribute or deploy their own version of AutoPlate. The documentation is available at https://philpalmer.github.io/AutoPlate/ and the source code at https://github.com/PhilPalmer/AutoPlate.

## Data Availability Statement

The datasets presented in this study can be found in online repositories. The names of the repository/repositories and accession number(s) can be found below: https://github.com/PhilPalmer/AutoPlate.

## Author Contributions

The project was conceived by DW who also provided the initial code. This was further developed into a web application by PP with feedback from DW, JR, KC, and GC. CH generated the example pMN data for Influenza H1N1 which was provided by GC. DW, JH, and NT supervised the project. PP wrote the manuscript with support from all authors. All authors contributed to the article and approved the submitted version.

## Funding

This work was supported in part by Innovate-UK PIVA grant, ref 105078 and the UKRI/NIHR grant nr. COV0170, the Humoral Immune Correlates to COVID-19 (HICC) consortium. PP is supported by a fellowship provided by The Open Philanthropy Project.

## Conflict of Interest

Authors DAW and JLH were employed by the company DIOSynVax.

The remaining authors declare that the research was conducted in the absence of any commercial or financial relationships that could be construed as a potential conflict of interest.

## Publisher’s Note

All claims expressed in this article are solely those of the authors and do not necessarily represent those of their affiliated organizations, or those of the publisher, the editors and the reviewers. Any product that may be evaluated in this article, or claim that may be made by its manufacturer, is not guaranteed or endorsed by the publisher.
