# Supplementary material for: AutoPlate: Rapid Dose-Response Curve Analysis for Biological Assays
Source: Front Immunol. 2022 Feb 10;12:681636. doi: 10.3389/fimmu.2021.681636 (PMC8866857; doi:10.3389/fimmu.2021.681636)
Supplement: Supplementary file 1 [file DataSheet_1.docx]

Supplementary Material

During testing the authors have found that due to technical error in some experiments, the virus only control wells are relatively low. This can negatively affect the curve fitting. Fortunately the types boxplot produced in autoplate can easily diagnose this issue. Figure 1b shows what this plot looks like when this error occurs, and the impact it has on the curves in figure 1d.

## Supplementary Figures


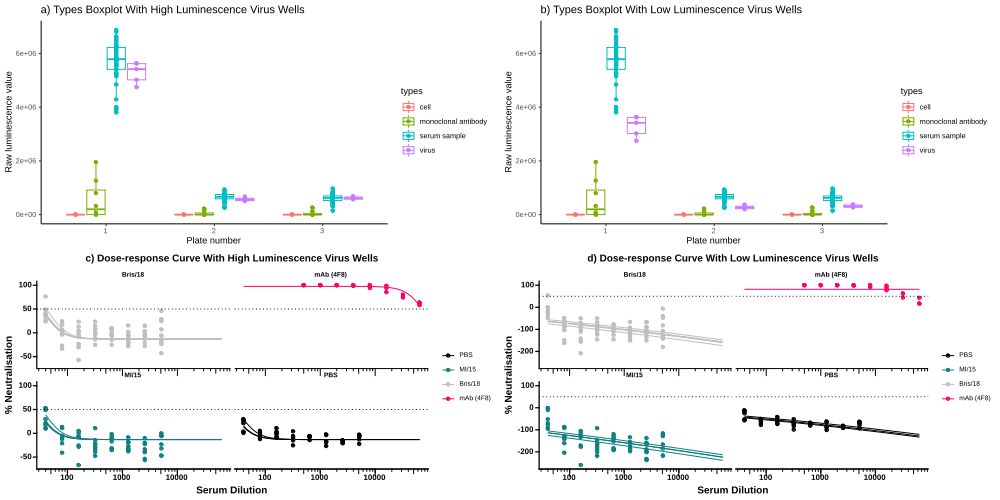


**Supplementary Figure 1.** Low luminescence values in virus only control wells negatively affects curve fitting. All of these plotted were generated using the first three plates of the example data and the luminescence values were artificially lowered for graphs b and d. Panels a) and b) show the Types Boxplot, the raw luminescence values split by plate and cell type, note the low virus luminescence in b). Panels c) and d) show the dose response curves fit to the data above. In panel c) the dose-response curves are smooth, fit the data well and flatten around 0% neutralisation. The curves in panel d) are very flat and fail to fit the data well, this is because of the low viral luminescence shown in panel b).
